# Current Knowledge on Genomic Profiling of Upper Tract Urothelial Carcinoma

**DOI:** 10.3390/genes12030333

**Published:** 2021-02-25

**Authors:** Elisa De Lorenzis, Giancarlo Albo, Fabrizio Longo, Carolina Bebi, Luca Boeri, Emanuele Montanari

**Affiliations:** 1 Urology Unit, Fondazione IRCCS Ca’ Granda Ospedale Maggiore Policlinico, 20122 Milan, Italy; giancarlo.albo@unimi.it (G.A.); fabrizio.longo@policlinico.mi.it (F.L.); carolina.bebi@unimi.it (C.B.); dr.lucaboeri@gmail.com (L.B.); emanuele.montanari@unimi.it (E.M.); 2 Department of Clinical Sciences and Community Health, University of Milan, 20122 Milan, Italy; 3 University of Milan, 20122 Milan, Italy

**Keywords:** genomics, upper tract urothelial carcinoma, urothelial cancer, targeted therapy

## Abstract

Recent research in next-generation sequencing characterized the genomic landscape of urothelial cancer. However, the majority of the studies focused on bladder cancer (BC). Upper urinary tract urothelial carcinomas (UTUC) and BC share some histological characteristics, but, considering the differences in terms of embryologic precursors, epidemiology, genetics, medical and surgical management and response to therapy, UTUC and BC should be considered as two distinct diseases. Our objective is to analyze through a literature search the latest updates and the current knowledge about the genomics of UTUC. We also evaluate genetic differences between BC and UTUC and the potential implications for systemic therapy. Molecular subtyping and variant histology and their correlation with response to chemotherapy were also explored. In summary, the most frequent genomic variations in UTUC included FGFR3, chromatin remodeling genes, TP53/MDM2 and other tumor suppressors/oncogenes. The genomics of UTUC, integrated with clinical data, could drive the selection of patients who could benefit from targeted therapy or off-label treatment. Routine implementation of tumor genomic characterization in UTUC patients should therefore be contemplated and evaluated prospectively.

## 1. Introduction

According to 2018 cancer statistics, 81,190 patients in the United States have been estimated to develop bladder cancer (BC) [1]. In men, BC is the fourth most common cancer and the eighth in terms of estimated deaths. Urothelial carcinomas can arise from either the lower urinary tract (bladder and urethra) or from the upper tract (renal pelvis, calyces and ureter). Upper urinary tract urothelial carcinomas (UTUCs) are less common (accounting for only 5–10% of all urothelial carcinomas) and the majority is represented by pyelocaliceal tumors [2]. Compared to BC, UTUC has a more aggressive clinical behavior and is more likely invasive at diagnosis [2].

The majority of UTUCs are pure urothelial cancers, however, a variant histology (VH) is reported in 13.4–24.2% of cases [3,4].

Aristolochic acid and smoking are established risk factors for UTUC; moreover, patients with Lynch syndrome are more affected by UTUC but not by BC [2]. The genomic characterization of UTUC can be useful not only to drive clinical management but also to recognize which tumors could be associated with Lynch syndrome. It has been documented that microsatellite instability and promoter methylation are more common in UTUC than in BC [5,6]. Possible explanations are the exposition to carcinogenic metabolites excreted in the urine or the diverse embryological origin of the upper urinary tract and bladder [7].

Radical nephroureterectomy (RNU) with bladder cuff excision is the standard treatment for patients with high-risk non-metastatic UTUC.

In this article, we analyze the latest updates and the current knowledge about the genomics of upper urinary tract urothelial carcinomas. We also evaluate differences between BC and UTUC and the potential implications for systemic therapy.

Eligible articles were identified through a literature search of PubMed, Scopus and Web of Science conducted in December 2020. The keywords used in the search were the following: upper tract urothelial carcinoma/cancer, urothelial carcinoma of the upper tract, UTUC, renal pelvis cancer/carcinoma, ureteral cancer/carcinoma, UTUC/upper tract urothelial carcinoma AND Lynch syndrome, aristolochic acid nephropathy, genomic AND UTUC/urothelial carcinoma of the upper tract, OR next-generation sequencing OR whole-exome sequencing.

We restricted our searches to publications from 2004 onward (the introduction of applicable next-generation sequencing platforms [8]). Additional papers were collected by cross-referencing the bibliography of the selected articles. Articles not in English, editorials, abstracts, non-human or in vitro studies were excluded.

Table 1 summarizes the major studies investigating the molecular characterization of UTUC included in this article.

## 2. Genomics of Upper Tract Urothelial Carcinoma

In 2015, Sfakianos et al. analyzed 83 low- and high-grade UTUCs [7]. The most frequently mutated genes were FGFR3 (54%), KMT2D (35%), KDM6A (34%), STAG2 (22%), CDKN2A (21%) and TP53 (18%).

In this study, the authors evaluated the association between genomic mutations and clinicopathological features. Recurrent activating mutations in FGFR3 were found in 95.6% of low-grade (LG) tumors. Mutations of TP53 were solely observed in high-grade (HG) tumors, whereas mutations in KDM6A and KMT2D were commonly detected in UTUCs of both low and high grade. 

Mutations in TP53, FGFR3, CREBBP, KMT2C and STAG2 were significantly associated with histological grade. Tumors presenting mutations of FGFR3, CREBBP and STAG2 were more frequently LG, whereas those with TP53 mutations were more frequently HG. T stage was also related to gene mutations. Tumors with FGFR3 mutations were usually pTa/pT1/pT2, whereas those with TP53, CCND1, ERBB2, ERBB3 and KRAS alterations were more frequently pT3/pT4.

These results are similar to those reported by Nassar et al: FGFR3 alterations were seen in 80% of LG-UTUC and TP53 mutations only in HG cancers [13].

Van Oers et al. demonstrated a similar FGFR3 mutations frequency in BC (48/100, 46%) and UTUC (71/147, 48%). FGFR3 was more frequently mutated in ureter specimens than in renal pelvis ones (59% vs. 39%) [20]. These alterations were associated with low-stage tumors and a less aggressive disease course in UTUC. Moreover, patients with pT2–pT4 UTUCs with FGFR3 mutations had a better survival.

An evaluation of the correlation between genetic alterations and anatomical UTUC origin was performed by Necchi et al. [19]. FGFR3 and HRAS mutations were more common in renal pelvis tumors, while the frequencies of KRAS and NRAS mutations were similar across anatomical sites (pelvis/ureter).

Moss et al. performed a comprehensive, integrated genomic analysis of UTUC on 31 patients (10 affected by ureteral cancer and 21 with renal pelvis neoplasm) [12]. At whole-exome sequencing, conducted on samples from 27 subjects, they found 2784 somatic mutations. At the analysis of the mutational profiles of UTUC, FGFR3 was found to be the most commonly mutated gene, in 74% of all tumors; the FGFR3 mutation rate rose to 92% in LG UTUC. 

Other identified altered genes in UTUC were: KMT2D (44.4%), PIK3CA (25.9%) and TP53 (22.2%). When the mutational characteristics of low- and high-grade tumors were compared, a higher incidence of mutations in the p53 signaling and greater genomic instability in HG tumors were shown to occur. 

Mutations of p53 have been identified in approximately 50% of all human cancers; expression of altered p53 has been detected in 30–60% of UTUC [21]. Numerous studies confirmed a correlation between p53 expression and lower survival rates and/or unfavorable parameters [22,23,24].

Audenet et al. prospectively sequenced 195 non-metastatic and metastatic UTUCs [17]. The most frequently mutated genes included FGFR3 (40%), KMT2D (37%), KDM6A (32%), TP53 (26%) and ARID1A (23%). Mutations were also analyzed considering histopathological variables. In patients with ≥ pT2 disease, alterations in the RTK/RAS pathway were less frequent, whereas alterations in TP53/MDM2 were more common. Activating alterations in FGFR3 and HRAS were less commonly detected in patients with higher stage disease. Conversely, the incidence of alterations in TP53 and MDM2 was considerably higher in patients with ≥ pT2 disease.

The link between genes mutations and bladder recurrence was evaluated, and alterations in FGFR3, KDM6A and CCND1 were found to be significantly associated with a higher risk of developing a subsequent bladder tumor, whereas TP53 alterations were associated with a lower risk.

Bagrodia et al. evaluated an association between genomic alterations in UTUC and adverse pathological and clinical outcomes in 83 patients with clinically localized disease treated with RNU [11]. They found that mutations of TP53/MDM2 are linked to worse clinicopathological outcomes, whereas FGFR3 alterations are associated with favorable outcomes. Mutations in TP53, TP53/MDM2 alteration and CCND1 alteration significantly increased the risk of death from disease, whereas FGFR3 mutation significantly decreased this risk. 

To differentiate adverse pathological and clinical outcomes. a risk score that takes into account TP53/ MDM2 and FGFR3 status (outlined in Table 2) was developed. This risk score was significantly associate with grade, stage and organ confined status. Moreover, higher risk scores worsened both the recurrence-free status and cancer specific survival (CSS).

A similar evaluation was performed to test the feasibility of genomic characterization on diagnostic ureteroscopic biopsy samples [18]. Specimen from ureteroscopy may be difficult to analyze due to the paucity of material (usually biopsies are performed with 1.9–2.4 Fr basket or 3–6 Fr cold cup forceps) [25]. 

In this study, 36/39 (92%) samples were considered adequate for sequencing and were compared to RNU specimens. TERT and FGFR3 were the two most frequently altered genes (64% of cases). As already reported in prior studies [7,12], chromatin remodeling genes including KMT2D (56%), KDM6A (47%), KMT2C (33%), ARID1A (31%) and CREBBP (31%) were commonly mutated. Alteration of TP53 was found in 25% of cases. A high level of concordance between matched biopsy and RNU specimens was found.

Overall, currently available studies regarding UTUC genomic mutations report comparable results. To summarize, the most frequently detected alterations include FGFR3, chromatin remodeling genes (e.g., KMT2D and KDM6A), TP53/MDM2 and other tumor suppressors/oncogenes such as CDKN2A or RAS. The frequency of FGFR3 alterations and TP53/MDM2 status is different across articles. This difference is due to the inconstant incidence of HG or primary and metastatic tumors analyzed in each article [8]. Table 3 summarizes the most common genetic mutations in UTUC divided by oncologic characteristics.

**Figure 1:**
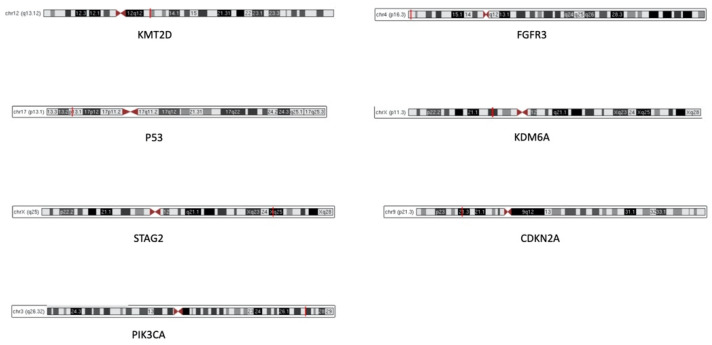
Cytogenetic location of the most frequent biomarkers in upper urinary tract urothelial carcinomas. Red lines indicate the exact location of each gene on the chromosomes.

Table 4 summarizes the most frequent biomarkers in upper tract urothelial carcinoma and their molecular functions, whereas Figure 1 depicts the cytogenetic location of the most frequent biomarkers in UTUC.

## 3. Genomics of Lynch Syndrome-Associated Upper Urinary Tract Urothelial Carcinomas

Lynch syndrome (LS) (i.e., hereditary non-polyposis colorectal cancer (HNPCC)) is an autosomal dominant multi-organ cancer syndrome resulting from germline mutations of mismatch repair (MMR) genes (MLH1, MSH2, MSH6 or PMS2). Cancers other than colorectal cancer associated with HNPCC are endometrial or ovarian cancer and cancer of the small bowel, biliary tract, stomach, pancreas, skin, brain or genitourinary tract [26]. UTUC is the third most frequent malignancy in LS [27,28].

Patients affected by HNPCC have a lifetime risk of developing UTUC 22 times higher than the general population [29]. UTUC had the highest overall lifetime risk (8.4%). Rates were 1.6-fold higher in males than in females and seven-fold higher in MSH2 than in MLH1 family members [30].

A 2018 study compared the genomics of a cohort of Lynch-associated UTUC with those from sporadic UTUC [15]. LS-UTUCs shown a significantly higher number of mutations per tumor, as expected in a microsatellite instability-associated malignancy. The most frequently mutated genes in the LS-UTUC group were KMT2D, CREBBP, ARID1A, SMARCA4, CIC, FAT1, FGFR3, FOXP1, KMT2C, NOTCH1 and NOTCH3.

The genomic mutations of LS-UTUC and sporadic UTUC were similar, although LS-UTUC presented with more alteration in genes such as KMT2D, CREBBP, ARID1A and SMARCA4. A few genes were somatic targets nearly exclusive to the LS cohort (i.e., CIC, FOXP1, NOTCH1, NOTCH3 and RB1). Both groups showed almost equal amounts of FGFR3 mutations, however LS-UTUC presented FGFR3 mutations that were mainly R248C, making it a possible biomarker for LS-UTUC patients. Of note, patients with LS were younger, had a lower exposure to tobacco and were more frequently affected by tumors located in the ureter.

Understanding the genomics characteristics of LS is useful to identify patients affected by LS-UTUC and to drive treatment, considering that hereditary cancers can often be misclassified as sporadic [31].

## 4. Genomics of Aristolochic Acid-Associated Upper Urinary Tract Urothelial Carcinoma

Aristolochic acids (AA) are nitrophenanthrene carboxylic acids found primarily in the Aristolochiaceae family of herbaceous plants [32]. Traditional Chinese medicine has used Aristolochiaceae plants for centuries; however, consumption of AA-containing herbs or plants has been associated with AA nephropathy and the development of UTUC.

The genomic of AA-associated UTUCs is different from that of sporadic tumors. DNA adducts exhibit mutagenic properties generating predominantly A:T to T:A transversions; moreover, endemic nephropathy-related UTUC showed an AA-specific mutational signature in the tumor suppressor gene TP53, dominated by A:T to T:A transversions, which is different from the mutational load of sporadic UTUC [9,33].

AA-associated UTUC is characterized by higher mutation burden as compared to sporadic cases. In particular, cancer promoting genes such as TP53, NRAS and HRAS were found to be frequently mutated in patients with nephropathy-related UTUC and next generation sequencing studies revealing that up to 83 cancer driver genes harbored signature mutations in these cohorts [10]. Of note, mutations were also highly prevalent in genes encoding chromatin re-modeling (such as MLL2 (62%), CREBBP (38%), KDM6A (15%) and members of the SWI/SNF family of proteins (SMARCA4: 15%; ARID1A, ARID1B and ARID2: 12%) [9], histone modification, transcriptional regulation, DNA damage response and DNA repair [10]. Further signatures identified included a signature related to age and a signature associated with the cytidine deaminase activity of APOBEC enzymes [10].

Differently from sporadic UTUC, FGFR3 mutations are rare in AA-induced tumors. It is reported that approximately 74% of UTUC cases in the United States showed FGFR3 mutations as compared to only 3% of nephropathic diseases [8,34].

From a clinical standpoint, AA-associated UTUC is diagnosed through deep sequencing of urinary sediment DNA, designed to detect a set of known oncogenic mutations and chromosomal aneuploidies [35]. Additionally, cell-free urinary DNA can be sequenced and specific mutational patterns identified [36].

The current standard of care for AA-associated UTUC is similar to that of sporadic disease. In a retrospective study in a population with endemic nephropathy, Cukuranovic et al. showed that patients with AA-associated UTUC were predominantly female, had poor renal function and presented with lower grade diseases [37]. Subsequently, Chen et al. analyzed oncologic outcomes of AA-related UTUC in 152 patients and revealed that contralateral recurrence free survival was significantly shorter in this group as compared to sporadic cases [38]. 

A recent study analyzed 942 UTUC patients treated with RNU confirmed that patients with AA exposure tended to be more frequently female and harbored lower tumor stage than those without exposure [39]. Of clinical importance, Authors found that AA exposure was associated with higher contralateral UTUC recurrence rates and greater intravesical recurrence but also higher CSS.

For advanced diseases, cisplatin-based chemotherapy is the treatment of choice, but this is limited by the frequent kidney impairment related to AA-nephropathy [40]. In this setting, since checkpoint inhibitors have stronger antineoplastic effects on tumors with high mutational burden, they are considered as promising therapeutic options for AA-induced urothelial carcinomas.

Overall, few studies have shown that AA-associated UTUC seems to be less lethal than sporadic cases; however, irrespective of the time since last exposure, patients with a history of AA exposure should be closely monitored due to their high risk of contralateral UTUC and bladder recurrence.

## 5. Differences between Bladder Cancer and Upper Urinary Tract Urothelial Carcinomas

Considering the differences in terms of embryologic precursors, epidemiology, genetics, medical and surgical management and response to therapy, UTUC and BC should be considered two different diseases and have been previously defined as “disparate twins” [41,42].

Differences in genomics can underlie clinical differences between the two tumors.

Sfakianos et al. compared the genomic profiles of high-grade UTUC (*n* = 59) and high-grade BC (*n* = 102) [7]. Interestingly, the profile of modifications in the UTUC and BC groups was similar, but mutation prevalence was different. FGFR3, HRAS and CDKN2B were more commonly mutated in UTUC, whereas TP53 and ARID1A were more commonly mutated in BC. A higher number of FGFR3-TACC3 fusions was detected in the UTUC group, whereas no RB1 mutations were found. The authors proposed a pattern in which low-grade tumors with FGFR3 and HRAS mutations may be more prone to progress to high-grade invasive disease when they occur in the upper tract than in the bladder.

Evaluating the differences in gene expression between UTUC and BC using microarray data, Sanford et al. did not reveal any differences between these two cancers [43]. However, when stratified by pathologic T stage, they found a differential clustering among pT3 tumors and significant gene expression differences in 81 genes. UTUC demonstrated lower levels of expression of genes enriched in HGF and TNF signaling pathways compared to BC. UTUC highly expressed genes associated with a luminal subtype. One of the genes mostly expressed in UTUCs was SLITRK6, a membrane with high levels in certain types of cancer, making it a target for antibody-drug therapy.

Audenet et al. concluded that, despite significant differences in prevalence of common genomic alterations in UTUC and BC, in patients with a history of both tumors, BCs and UTUCs are always clonally related [17].

In a recent study investigating the mutational and transcriptional profiles of commonly mutated genes in limited numbers (*n* = 92) of UTUC and BC, the authors concluded that BC and UTUC shared common molecular features [14]. The frequency of mutations was, in fact, not significantly different between BC and UTUC. In the UTUC cohort, the most frequent somatic mutations were TP53 (71.0%), KDR (35.5%) and TERT (16.1%); no RB1 mutations were detected. 

Interestingly, Nassar et al. found that the mutational landscape of low-grade UTUC was similar to that of LG non-muscle invasive BC with a prevalence of KDM6A, STAG2 and FGFR3 alterations [13].

One of the largest cohorts investigating the molecular profile of UTUC and BC has demonstrated that urothelial cancer had a landscape of 70% “possibly targetable/actionable” genomic alterations [19]. TERT, TP53 and CDKN2A were the most frequently mutated genes, but TERT and TP53 were less common in UTUC. As previously reported, FGFR3 and HRAS alterations were more common in UTUC.

## 6. Molecular Subtypes

In 2017, the Cancer Genome Atlas (TCGA) study classified urothelial BC into five molecular subtypes: luminal-papillary, luminal-infiltrated, luminal, basal/squamous and neuronal [44]. However, TCGA did not include UTUC. Consequently, the knowledge of the key biological features of UTUC is partial. 

Moss et al. analyzed 31 UTUC samples and defined four molecular subtypes through RNA sequencing [12]. This classification correlates with clinical and histopathological features.

A comparison between these data and the TCGA dataset revealed that Cluster 1 (*n* = 5) is comparable to the BC luminal subtype, Cluster 2 (*n* = 6) is closer to the basal subtype and Cluster 4 (*n* = 9) has a high frequency of upregulated immune checkpoint genes. 

**Figure 2:**
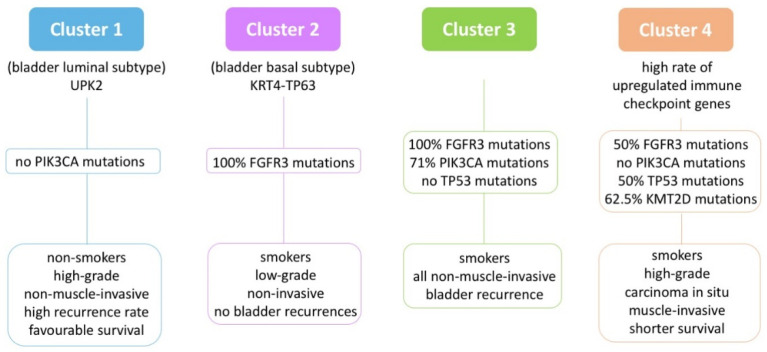
RNA sequencing UTUC molecular subtypes and their correlation with clinical variables. Adapted from Moss et al. [12].

Figure 2 depicts a schematic representation of the characteristics of the four subtypes. 

Robinson et al. performed an integrated analysis of whole-exome and RNA sequencing of high-grade primary UTUC [16]. They found that most UTUC are luminal-papillary (20/32, 62.5%), with the majority of remaining UTUCs also exhibiting a luminal expression profile. In the TCGA cohort, only the 27.3% (35/128) of cases were luminal-papillary urothelial BC [44].

McConkey et al. evaluated an association between gene expression profiling, clinical outcomes and urothelial cancer subtypes in patients treated with neoadjuvant chemotherapy (NAC) [45]. In this cohort, 26.7% of patients were affected by HG-UTUC; the most common subtype was luminal. Overall, in the NAC setting, patients with basal tumors had better survival compared to the luminal and p53-like subtypes.

## 7. Variant Histology

A variant histology can be defined as nonpure urothelial cancer, including urothelial cancer with VH and pure VH. Any type of histology that may be found in the bladder may also, theoretically, be found in the upper urinary tract. The identification of VH is crucial for accurate diagnosis, prognosis and to tailor the correct therapeutic strategy. Previous studies analyzing differences between pure BC and BC with variant histology showed that BC with VH is usually associated with more aggressive features and worse clinical outcomes [46,47,48]. It has been demonstrated that small-cell bladder carcinoma is the most aggressive, but also the most chemosensitive subtype of VH. Other VHs appeared to be more chemoresistant than pure UC [46].

Table 5 summarizes the VH of UTUC, according to the findings from Rink et al. [4].

A recent metanalysis investigating the prognostic value of VH in patients with UTUC revealed that VH was significantly associated with poorer cancer specific, overall and recurrence-free survival [3]. 

The most common VH of urothelial cancer is the squamous differentiation, that occurs in up to 20–40% of UTUCs [49]. In the squamous differentiation, high levels of PDL-1 expression have been found [50].

In BC, the basal subtype includes nearly all the tumors with squamous differentiation [37,49].

The micropapillary variant of upper tract cancers is usually aggressive, with advanced stage at diagnosis, lymphovascular invasion and a high tendency to metastasize [51,52]. In this disease, ERBB2 mutations and HER2 overexpression are more common than in pure urothelial cancer [53].

Plasmacytoid UTUC has been investigated in some case reports [54,55]: it is a rare tumor characterized by poor prognosis with metastasis at presentation in the retroperitoneum. In bladder plasmacytoid cancers, it has been found that PD-1 and PDL-1 are usually not expressed [56]. Moreover, plasmacytoid BC is associated with a luminal expression profile [57].

The nested variant of urothelial carcinoma is a rare histologic type of urothelial carcinoma, with the majority of cases involving the bladder. Few cases have been documented in the upper tract, and the optimal treatment of this VH has not been standardized. In all of the few cases reported in the literature, the selected treatment was RNU and only in one case an adjuvant chemotherapy was performed. The authors concluded that the nested variant of UTUC should be considered as a high-grade cancer despite the presence of low-grade features, similar to its counterpart arising in the urinary bladder [58].

Sarcomatoid carcinoma of transitional cell origin in the upper urinary tract is rare and should be differentiated from sarcomatoid renal cell carcinoma.

Compared to UTUC, sarcomatoid urothelial carcinoma seems to be more malignant and with a poor prognosis. Usually, the disease is metastatic or advanced when first discovered. Considering all reports, in the case of upper urinary tract sarcomatoid carcinoma, life-expectancy is limited [59,60,61].

A study exploring the molecular characterization of sarcomatoid carcinoma of the upper urinary tract concluded that a moderate to strong EGFR expression was demonstrable in 75% of cases, suggesting that an anti-EGFR therapy may be investigated as an adjuvant therapeutic strategy for this rare neoplasm [62].

Neuroendocrine UTUC includes small cell carcinoma, large cell carcinoma and those with mixed patterns. 

Since 1980, fewer than 40 cases of primary upper urinary tract small cell tumors have been reported [63,64,65]. In the majority of cases, small cell carcinoma coexists with another malignant histologic component, including urothelial cancer [65]. Usually, in this type of cancer, co-alterations in TP53 and RB1 resulting in loss of function of both genes were detected. In BC setting, it is unclear if true small cell carcinoma is related to the neuronal subtype, defined by the TCGA [66].

Due to aggressive features and resistance to therapy, a multimodal approach has usually been used for the treatment of upper tract small cell carcinoma (surgery, external radiotherapy and (neo) adjuvant chemotherapy). Limited data seem to suggest that cisplatin-based neoadjuvant chemotherapy prolongs disease-free survival [67].

As borrowed from BC studies, the association of VH and their defined mutation patterns could be useful in exploring targeted therapeutic approaches based on specific molecular pathways [68].

Due to the rarity of the VH, their management represents a diagnostic challenge considering the lack of a standardized approach. The information regarding the prognostic value of VH in patients with UTUC can be useful to counsel and identify patients for adjuvant therapy and for more rigorous follow-up protocol.

## 8. Implications for Prognosis Predication after Radical Nephroureterectomy or Kidney-Sparing Surgery

The gold standard treatments for localized UTUC are radical nephroureterectomy with bladder cuff excision for high-risk tumors and kidney sparing surgery with endoscopic ablation or segmental ureterectomy for low-risk cases.

Preoperative predictors of worse prognosis include age at diagnosis, African American ethnicity, exposure to tobacco consumption, multifocal and/or ureteral tumor location, surgical delay, obesity and pre-treatment-derived neutrophil–lymphocyte ratio [2].

Postoperative predictors of worse prognosis include high tumor stage and grade, lymph node involvement, lymphovascular invasion, positive surgical margins, extensive tumor necrosis, sessile growth pattern architecture and concomitant carcinoma in situ [2].

Recently, biomarkers associated with biologically aggressive disease and the prognosis of patients with UTUC have been investigated.

The explored biomarkers are involved in cell cycle regulation, cell growth, proliferation and differentiation, signal transduction, angiogenesis, apoptosis and cell adhesion.

Rey et al. were the first to evaluate the prognostic role of proteins involved in cell-cycle regulation in patients with UTUC [22]. p53 immunostaining was performed on paraffin embedded tissue from 83 patients treated between 1975 and 1993. p53 overexpression was significantly associated with tumor aggressiveness and patient survival. The prognostic value of p53 was recently confirmed in a systematic review and meta-analysis: 514 patients from seven studies were included and statistically significant differences in disease-free (DFS), CSS and overall survival (OS) were found, suggesting that p53 is an independent prognostic factor in UTUC [69].

In contrast, a recent review analyzed 24 papers, in five of which multivariate analysis demonstrates that p53 expression is of independent prognostic significance in UTUC, all of which contained potential statistical bias [21]. The authors concluded that available data do not support p53 as an independent prognostic marker in UTUC.

Cyclins proteins were investigated in patients affected by UTUC. Liang et al. [70] studied 340 patients with localized disease treated by ureterectomy or RNU and found that nuclear expression of Cyclin A was associated with a poor disease-specific survival (DSS) (*p* = 0.0035) and metastasis-free survival (MFS) (*p* = 0.0015) in the univariate analysis but was not significative in the multivariate analyses. The authors demonstrated that patients with elevated HuR cytoplasmic expression, an RNA-binding protein that modulates the expressions of cyclin A, had better DSS if adjuvant chemotherapy was performed (*p* = 0.015). Van Oers et al. [20] reported that FGFR3 mutations correlated with low-stage tumors and better survival in patients with UTUC.

Wang et al. [71] investigated the expression level of KDM6A in 108 surgically resected UTUC samples. The authors found that lower KDM6A expression was significantly associated with a higher tumor grade and shorter CSS and DFS times (*p* = 0.023 and *p* = 0.033, respectively).

The study of Singla et al. [72] included 376 patients who underwent RNU for high-grade UTUC from 1990 to 2008. The authors found that on univariate analysis increased EZH2 expression was a significant predictor for inferior recurrence free survival (*p* = 0.033), CSS (*p* = 0.003) and OS (*p* < 0.001). On multivariate analysis, EZH2 remained a significant predictor of worse CSS (HR 1.99 (95% CI: 1.21–3.27), *p* = 0.007) and OS (HR 1.54 (95% CI: 1.06–2.24), *p* = 0.024).

Miyakawa et al. [73] studied the expression of STAG2 in 171 patients with UTUC who underwent RNU. They found that patients with STAG2 loss tended to have good prognosis in both MFS (*p* = 0.16) and CSS (*p* = 0.38), but neither showed statistical significance in either the uni- or multivariate analysis. However, STAG2 loss was significantly associated with worse clinical outcome in UTUC with high Ki-67 proliferation indexes, but not in UTUC with low Ki-67 expression.

Despite the growing body of evidence on tissue-based markers in UTUC in the past several decades, further development is needed for their use in daily practice.

## 9. Implications for Systemic Therapy

Considering staging limitations (endoscopic biopsy provide information regarding grading but not T-stage), the standard therapy for high grade tumors is generally RNU. However, radical surgery may represent overtreatment for patients with lower stage disease. On the other hand, patients with tumors that may benefit from NAC are not usually identified before surgery.

Systemic chemotherapy is the standard approach in patients with locally advanced or metastatic urothelial diseases [2]. Due to the development of renal insufficiency after RNU, many patients are not eligible for adjuvant platinum-based chemotherapy [74,75].

These conditions make multimodal treatment of UTUC challenging.

A molecular profiling approach to UTUC could be useful in the preoperative setting, as proposed by Bagrodia et al. [11]. For example, patients with altered TP53/MDM2 may be considered for adjuvant chemotherapy or enrollment in clinical trials. Moreover, a genomic characterization of tumor biopsy samples may help to select high-risk patients who could benefit from NAC and screen low-risk patients who could be managed with a kidney-sparing approach [18].

Recently, immunotherapy has been investigated as a promising treatment strategy for several malignancies, especially in BC in a neoadjuvant setting [76]. However, the role of immunotherapy has been evaluated both in the first-line setting for cisplatin-ineligible urothelial cancer patients (affected by BC and UTUC) and in the second-line setting, in platinum-pretreated patients. However, limited data about UTUC patient outcomes are available in the literature [77,78,79,80,81,82,83]. Consequently, the prognostic implication of programmed death-ligand 1 expression remains unclear in patients with UTUC.

Current knowledge on immune checkpoint inhibitors applied in the adjuvant treatment of urothelial cancer is summarized in Table 6.

In summary, patients with UTUC represent a small portion of the patients affected by urothelial cancer studied in the main trials; nevertheless, available data are encouraging [84].

**Figure 3:**
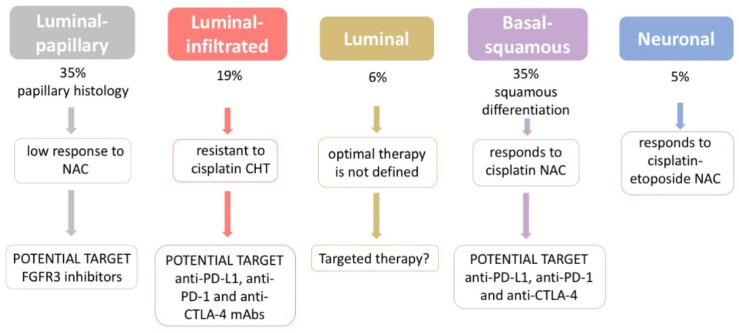
Adapted scheme of subtype-stratified therapeutic approach proposed by Robertson et al. [44].

In bladder cancer, a subtype-stratified therapeutic approach has been hypothesized [44], as summarized in Figure 3.

No conclusions can be drawn regarding the distribution of molecular subtypes in UTUC, but some studies concluded that most UTUCs were of the luminal subtype with an associated T-cell-depleted microenvironment [8,16,43].

In luminal subtypes, the presence of FGFR signaling or ERBB2 expression suggest a potential role for targeted agents, while ERBB2 expression may help in selecting for chemosensitive luminal urothelial cancer [85]. 

The molecular subtyping proposed by Moss et al. suggests a role for FGFR3 inhibitors in both LG- and HG-UTUC [12,86]; moreover, immune checkpoint inhibitors might be effective in the cohort of patients belonging to Cluster 4 (Figure 2).

Considering the expression of SLITRK6 in UTUC [43], this marker is a potential target for patients with advanced upper urinary tract cancer. Interestingly, an antibody to the SLITRK6 protein linked with a cytotoxic agent monomethyl auristatin E (AGS15E) has been developed and is in phase I Clinical Trials for the treatment of advanced urothelial cancer (NCT 01963052) [87].

## 10. Conclusions

Genomic characterization of UTUC, integrated with clinical information, could drive the selection of patients who could benefit from targeted therapy or off-label treatment. 

Routine implementation of UTUC genomics analysis should therefore be contemplated and evaluated prospectively.

The latest genomic studies exposed the molecular landscape of UTUC and contributed to identify several targetable gene alterations that could become object of investigation for targeted therapies, either in combination with cytotoxic agents or as single agents.

## Figures and Tables

**Table 1:** Major studies assessing molecular characterization of UTUC.

Author	Year	Method	*n*. UTUC	*n*. Bladder Cancer	Characteristics of UTUC Patients
Hoang [9]	2013	whole exome sequencing	19	0	Patients with documented exposure to AA Pathological data not available
Sfakianos [7]	2015	NGS array (300 genes)	83	102 (all HG)	All UTUC patients treated with RNU Female patients 33.7%; median age 68 (63–75) years Tumor grade: 71.1% HG-UTUC No predominant variant histologies were included Tumor stage: 20.5% pN+; 57% Pt ≥ 2
Castells [10]	2015	low-coverage whole-exome sequencing	17	0	Urothelial tumors from 15 patients with aristolochic acid nephropathy Pathological data not available
Bagrodia [11]	2016	NGS array (300 genes)	82	0	All patients treated with RNU Female patients 34.1%; median age 68 (38–68) years Tumor grade: 72% HG-UTUC Tumor stage: 20.7% pN+; 46.3% pT ≥ 2 Tumor site: renal pelvis (52.4%), pelvis and ureter (31.7%), ureter (15.9%) 8.5% received NAC
Moss [12]	2017	whole exome sequencing	31	0	Patients treated with endoscopic biopsy or surgical resection Female patients 32%, median age 74 (68–80) years Tumor grade: 55% HG-UTUC No variant histology was present Tumor stage: 32% pT ≥ 2 Tumor site: ureter (32.2%), renal pelvis (67.8%)
Nassar [13]	2018	targeted exome sequencing (237 genes)	65	407 (31% HG and 49% MIBC)	Female patients 29.2%; median age 68 (45–88) years in HG-UTUC Tumor grade: 84.6% HG-UTUC Variant differentiation: 18.5% Tumor stage: 78.5% pT ≥ 2, 23.1% pN+, 12.3% were metastatic 16.9% received prior chemotherapy treatment
Lee [14]	2018	NGS with Ampliseq (50 genes)	31	61	All UTUC patients treated with RNU, LND was performed in only 48.4% of UTUC patients Female patients 29%, median age 65 (50–79) years Tumor grade: 67.7% HG-UTUC Tumor stage: 96.8% pT ≥ 2, 29% pN+ 3.2% received NAC
Donahu [15]	2018	MSK-IMPACT assay (341 genes)	17	0	Patients with Lynch syndrome Female patients 47%; median age 61 (53–66) years Tumor grade: 71% HG-UTUC Tumor stage: 45% pT ≥ 2 Tumor site: renal pelvis (53%), ureter (47%)
Robinson [16]	2019	whole-exome sequencing and RNA sequencing	37	0	Tumor grade: all HG-UTUC 84.3% of the UTUC tumors clustered with the luminal subtype
Audenet [17]	2019	NGS platform	195	454 (94% HG)	Female patients 38%; median age 67.1 (58.1–74.5) years Tumor grade: 85% HG-UTUC Variant differentiation: 12% Tumor stage: 42% pT ≥ 2; 14% were metastatic Tumor site: renal pelvis (79%), ureter (21%)
Bagrodia [18]	2019	hybridization-based exon capture assay (410 genes)	36 (biopsy) 130 (RNU)	0	Tumor grade: 34% HG-UTUC in the biopsy cohort, 17% in the RNU group
Necchi [19]	2020	hybrid capture-based comprehensive genomic profiling	479	1984	Female patients 38%, median age 68 (61–75) years Source of the analyzed tumor: 61% primary tumor, 18% visceral metastasis, 8.4% lymph node metastasis, 12.5% unknown All primary tumor samples were of high grade Tumor site: renal pelvis (66%), ureter (34%)

Legend: UTUC, upper tract urothelial carcinoma; AA, aristolochic acid; NGS, next-generation sequencing; RNU, radical nephroureterectomy; HG, high-grade; NAC, neoadjuvant chemotherapy; MIBC, muscle-invasive bladder cancer; LND, lymph node dissection.

**Table 2:** Risk score from Bagrodia et al. [11] using TP53/ MDM2 and FGFR3 status to discriminate between adverse pathological and clinical outcomes.

Score	TP53/MDM2 Status	FGFR3 Status
0	normal	altered
1 2	Normal altered	Normal normal

**Table 3:** Summary of the most common genetic mutations in UTUC divided by oncologic characteristics.

Oncologic Characteristic	Mutational Landscape
Low-grade tumor	↑ FGFR3 mutations, ↓ TP53/MDM2 mutations
High-grade tumor	↑ mutations genes p53 signaling (TP53, ATM, ATR) ↓ FGFR3 mutations
Higher stage	↓ alterations RTK/RAS pathway ↓ activating alterations of FGFR3 and HRAS ↑ TP53/MDM2 alterations ↑ TP53, ATM, ATR mutations
Metastatic disease	↑ TP53 and MDM2 mutations < FGFR3 mutations than in primary tissue

↑ increased, ↓ reduced, < lower.

**Table 4:** Molecular functions of the most frequent biomarkers in upper urinary tract urothelial carcinomas.

Biomarker	Description	Function	Cytogenetic Location
FGFR3	Fibroblast Growth Factor Receptor 3	Transmembrane tyrosine kinase protein, member of the fibroblast growth factor receptor family. Involved in MAP kinase signaling pathway and AKT1 signaling pathway	4p16.3
KMTD2	Histone-lysine N-methyltransferase 2D	Histone methyltransferase that methylates ASCOM protein complex, transcriptional regulator of the beta-globin and estrogen receptor genes	12q13.12
TP53	Tumor Protein P53	Tumor suppressor; induces growth arrest or apoptosis depending on the physiological circumstances and cell type	17p13.1
KDM6A	Lysine demethylase 6A	Histone demethylase that specifically demethylates “Lys-27” of histone H3, thereby playing a central role in histone code	Xp11.3
STAG2	Stromal antigen 2	Chromatin binding, component of cohesin complex required for the cohesion of sister chromatids after DNA replication	Xq25
CDKN2A	Cyclin Dependent Kinase Inhibitor 2A	Regulate 2 cell cycle regulatory pathways: the p53 pathway and the RB1 pathway	9p21.3
PIK3CA	Phosphatidylinositol-4,5-Bisphosphate 3-Kinase Catalytic Subunit Alpha	Lipid kinases responsible for coordinating a diverse range of cell functions including proliferation and survival. Involved in AKT and mTOR pathways	3q26.32

**Table 5:** Frequency of histological variants in UTUC, adapted from Rink et al. [4].

Urothelial Carcinoma Histology	Frequency
Pure Variant histology - Squamous - Glandular - Sarcomatoid - Micropapillary - Small cell (neuroendocrine) - Plasmacytoid - Multiple	75.8% 24.2% 9.9% 4% 2.4% 1.9% 1.9% 0.2% 3.9%

**Table 6:** Studies assessing immune checkpoint inhibitors in the first- and second-line treatment of patients with locally advanced/metastatic/unresectable urothelial cancer.

Author	Year	Drug	Setting	Patients	Results
Galsky [79] IMvigor130 NCT02807636	2020	Atezolizumab plus platinum-based CHT (group A), atezolizumab monotherapy (group B) or placebo plus platinum-based CHT (group C)	Locally advanced or metastatic UC	Group A - LTUC 322 (71.4%) - UTUC 123 (27.3%) - other 8 (1.3%) Group B - LTUC 271 (74.9%) - UTUC 89 (24.6%) - other 2 (0.5%) Group C - LTUC 298 (74.5%) - UTUC 100 (25%) - other 2 (0.5%)	Addition of atezolizumab to platinum-based CHT as first-line treatment prolonged PFS in patients with metastatic UC Median follow up 11.8 (6.1–17.2) months
Powles [81] IMvigor211 NCT02302807	2018	Atezolizumab (anti- PD-L1) versus CHT (vinflunine, paclitaxel, docetaxel)	Platinum-treated locally advanced or metastatic UC	#ATZ group: - LTUC 333 (71.3%) - UTUC 126 (27%) - other 8 (1.7%) CHT group: - LTUC 347 (74.8%) - UTUC 110 (23.7%) - other 7 (1.5%)	OS did not differ significantly between groups; OS seems to be better with CHT in renal pelvis group [HR 1.32 (0.50–3.48)] Safety profile for atezolizumab was favorable compared with CHT Median follow up 17.3 (0–24.5) months
Patel [80] JAVELIN Solid Tumor NCT01772004	2018	Avelumab (anti-PD-L1 IgG1 antibody)	Metastatic UC after platinum failure	- LTUC 191 (77%) - UTUC 58 (23%)	Objective response: - 11% in UTUC - 18% in LTUC Median follow up 9.9 (4.3–12.1) months
Balar [78] IMvigor210 NCT02108652	2017	Atezolizumab (anti- PD-L1)	First-line in locally advanced or metastatic UC, cisplatin ineligible patients	- LTUC 85 (71%) - UTUC 33 (28%)	Objective response: - 39% in UTUC - 17% in LTUC Most frequent responses in luminal II subtype and in higher tumor mutation load Median follow up 17.2 (0.2–23.5) months
Balar [77] KEYNOTE-052 NCT02335424	2017	Pembrolizumab (anti-PD-1 antibody)	First-line in locally advanced or unresectable or metastatic UC, cisplatin ineligible patients	- LTUC 300 (81%) - UTUC 69 (19%)	Tumor response: - 22% in UTUC - 28% in LTUC PD-L1-expression cut-off 10% associated with a higher response Median follow up 5 (3.0–8.6) months
Bellmunt [82] KEYNOTE-045	2017	Pembrolizumab (anti-PD-1 antibody) versus CHT (paclitaxel, docetaxel, vinflunine)	Advanced UC that recurred or progressed after platinum-based CHT	PMZ group: - LTUC 232 (85.9%) - UTUC 38 (14.1%) CHT group: - LTUC 234 (86.3%) - UTUC 37 (13.7%)	Longer OS and lower rate of treatment-related adverse events in PMZ group
Rosenberg [83] NCT02108652	2016	Atezolizumab (anti- PD-L1)	Locally advanced and metastatic UC that progressed after platinum-based CHT	- LTUC 235 (75.8%) - UTUC 65 (21%) - other 10 (3.2%)	Objective response *: - 17% in bladder - 7% in renal pelvis - 9% in ureter Significantly higher response in the luminal II subtype and in in higher tumor mutation load

Legend: PD-L1, programmed death-ligand 1; UC, urothelial cancer, CHT, chemotherapy; ATZ, atezolizumab; LTUC, lower tract (bladder, urethra) urothelial carcinoma; UTUC, upper tract (renal pelvis, ureter) urothelial carcinoma; PFS, progression-free survival; OS, overall survival; PMZ, pembrolizumab. # Intention-to-treat population; * by RECIST v1.1.
